# Right Ventricular Hydatid Cyst in a Young Adult: A Rare Case From the UAE

**DOI:** 10.1016/j.jaccas.2025.104337

**Published:** 2025-07-23

**Authors:** Khalid Elhaj, Tasnima Tayb, Ahmed Bafadel, Amritha Omprakash, Zainab Hashmi, Gopal Bhatnagar

**Affiliations:** a Al Ain Hospital, Abu Dhabi Health Services Company (SEHA), Abu Dhabi, United Arab Emirates; b Heart, Vascular and Thoracic Institute, Cleveland Clinic Abu Dhabi, Abu Dhabi, United Arab Emirates; c Cleveland Clinic Abu Dhabi, Abu Dhabi, United Arab Emirates

**Keywords:** cardiac mass, echinococcus cyst, hydatid cyst, right ventricle

## Abstract

**Background:**

Cystic echinococcosis, or hydatid cystic disease, is a globally prevalent zoonotic infection that typically affects the liver or lungs.

**Case Summary:**

A 28-year-old man residing in the UAE presented with chronic hemoptysis, melena, and weight loss. Chest radiography revealed multiple bilateral lung nodules, and a subsequent surgical lung biopsy confirmed necrotizing granulomatous inflammation, suggestive of a chronic inflammatory process. Although initially delayed, transthoracic echocardiography detected a cystic mass (2.4 × 2.0 cm) and a solid mass (1.0 × 0.9 cm) on the right ventricular free wall, with no impact on right ventricle function. Albendazole treatment was initiated based on positive hydatid serology, followed by cardiac magnetic resonance imaging and surgical excision of the cardiac masses.

**Discussion:**

Cardiac involvement in cystic echinococcosis accounts for only 0.5% to 2% of cases, with right ventricular localization reported in approximately 10% of those. This case highlights the diagnostic complexity of echinococcosis and underscores the importance of comprehensive evaluation and individualized management, particularly in young adults.

## Graphical Abstract

**Figure:**
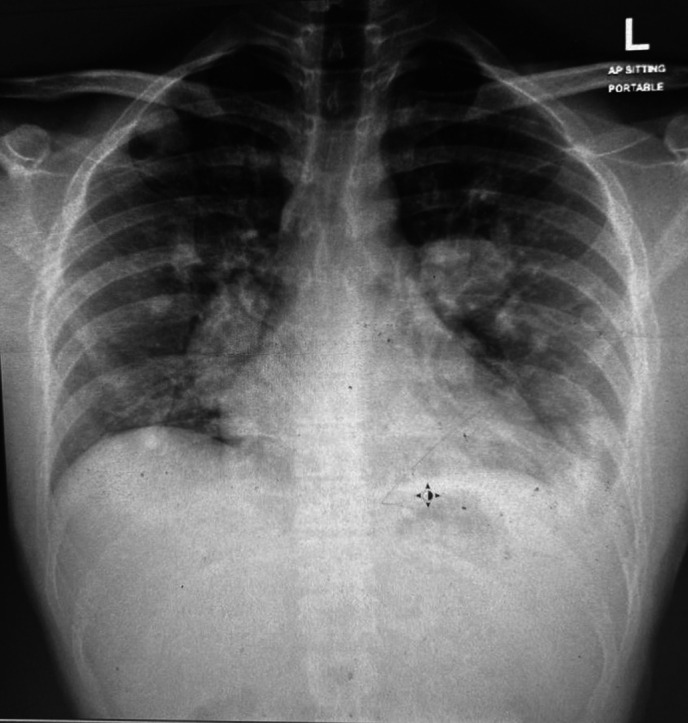

## History of Presentation and Past Medical History

A 28-year-old previously healthy man first presented to an external hospital in January 2024 with hemoptysis, melena, and a 3-kg weight loss. He had multiple prior hospital visits for similar complaints. He arrived to the UAE in 2021 and worked as a security guard. Born and raised in India, he denied any contact with animals in his home country and reported no recent travel. He was vitally stable, with a temperature of 36.6 °C, blood pressure of 105/68 mm Hg, heart rate of 74 beats/min, respiratory rate of 18 breaths/ min, and oxygen saturation of 99% on room air.

**Take-Home Messages**

• Delayed use of transthoracic echocardiography contributed to a missed early suspicion of cardiac involvement.
• Despite extensive imaging and surgical biopsy, confirmation of helminthic infection was ultimately achieved through serologic testing for parasitic antibodies.
• Surgical resection remains the mainstay of treatment for cardiac echinococcosis because cyst rupture can be fatal.

## Differential Diagnosis

The differential diagnosis included primary lung cancer and pulmonary metastases from an extrapulmonary malignancy.

## Investigations

**Figure 1:**
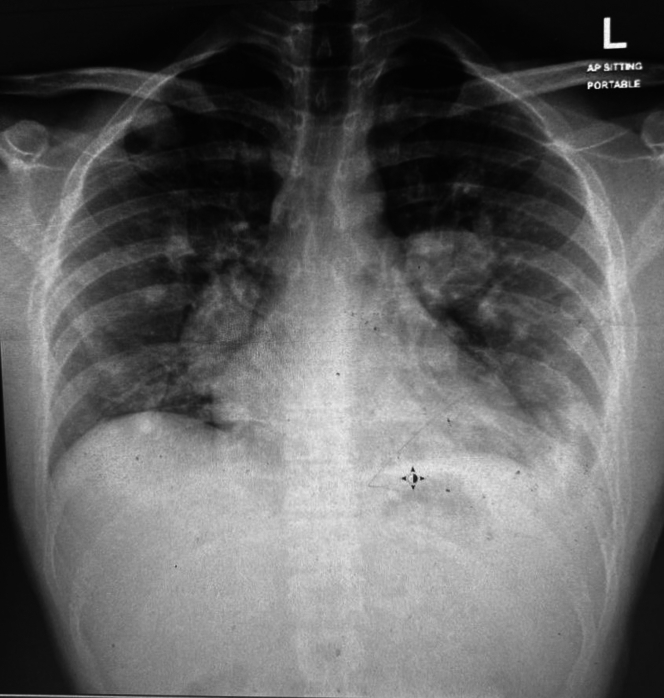
Chest Radiograph Showing Multiple Bilateral Lung Nodules

Owing to suspicious bilateral chest nodules on chest radiography (Figure 1), the patient underwent a computed tomography (CT)-guided surgical lung biopsy. The biopsy revealed a necrotizing granulomatous inflammatory process with focal laminated eosinophilic material, suggestive of echinococcus helminthic infection, with no evidence of malignancy.

**Figure 2:**
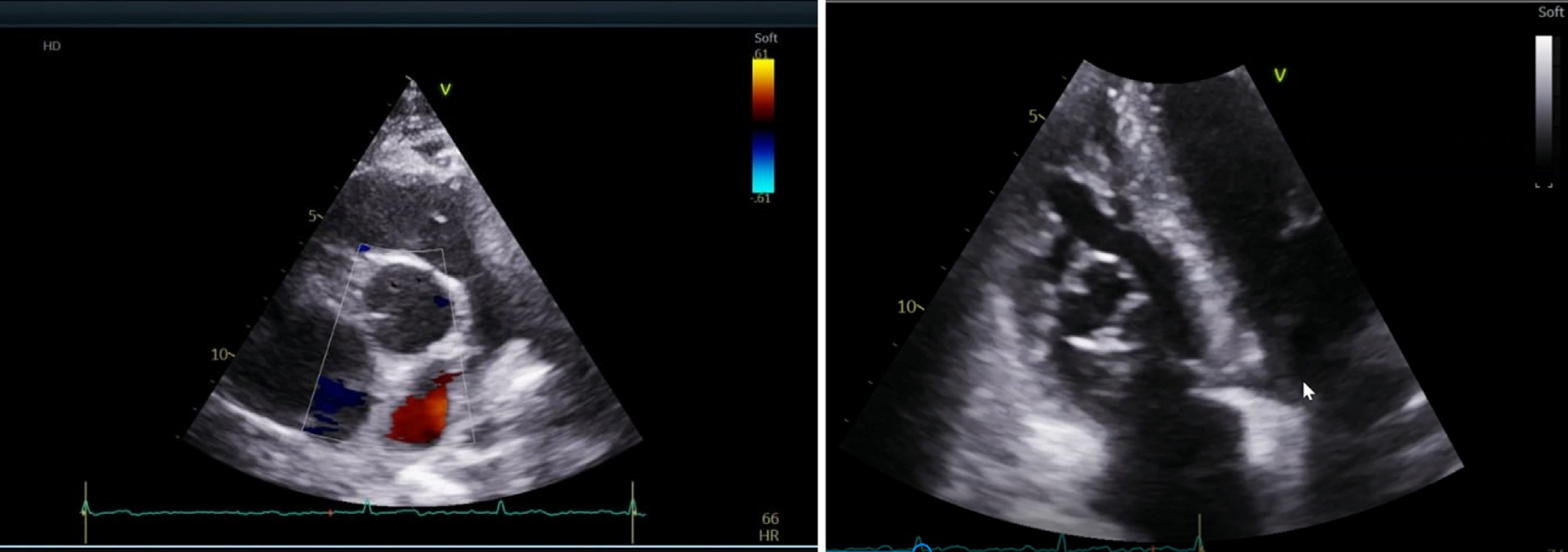
TTE Revealing RV Cystic Masses Attached to the RV Free Wall (A and B) TTE (aortic valve in short-axis parasternal view) showing cystic masses attached to the RV free wall. The left ventricle appeared normal with preserved systolic and diastolic function; the ejection fraction was 60%. A large mass measuring 2.4 × 2.1 cm was visualized in the RV, attached to the RV free wall just below the lateral tricuspid valve annulus. A mobile component was noted on the mass surface. Differential diagnoses included secondary cardiac masses. No significant valvular abnormalities were observed. RV = right ventricle; TTE = transthoracic echocardiography.

**Figure 3:**
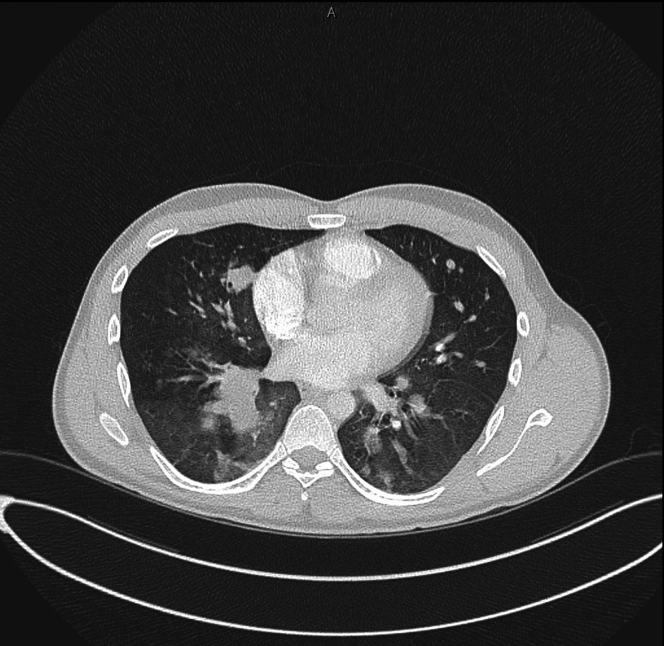
CTPA Demonstrating a RV Hydatid Cyst and Features of Disseminated Hydatid Disease Chest CT demonstrating a large hydatid cyst in the RV, measuring approximately 32 × 54 mm. A large intraluminal hydatid clot is seen in the right lower pulmonary artery, with an adjacent subsegmental pulmonary infarct in the right lower lobe. A hypertrophied right bronchial artery, arising from the aorta, runs parallel to the occluded pulmonary artery. Innumerable low-attenuation cystic-like nodules are scattered throughout both lungs, consistent with disseminated hydatid disease. No evidence of parasitic involvement was observed in the vertebrae, liver, or brain on subsequent CT scans of the abdomen and head. CT = computed tomography; CTPA = CT pulmonary angiogram; RV = right ventricle.

**Figure 4:**
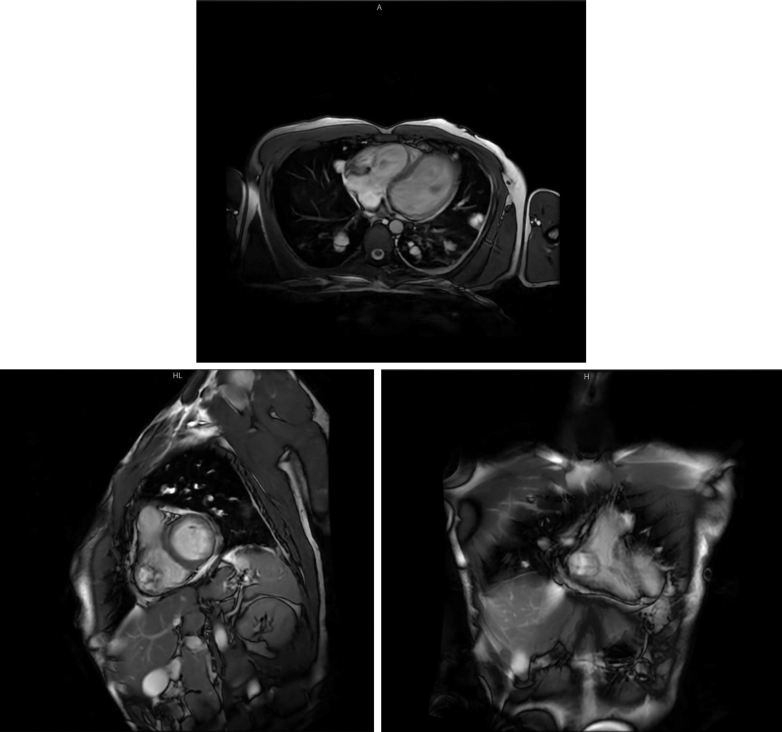
CMR Demonstrating a Benign Multicystic Lesion in the RV (A-C) Cardiac mass revealed a multicystic lesion measuring 26 × 30 mm, attached to the RV free wall. The lesion appeared hyperintense on both T1- and T2-weighted images. There was no evidence of thrombus, no perfusion on first-pass imaging, and no late gadolinium enhancement—features consistent with a benign, avascular lesion. RV function was preserved. The left ventricle demonstrated normal indexed volumes, wall thickness (maximum ∼8 mm at the basal anteroseptum), and ejection fraction. No regional wall motion abnormalities were observed at rest. There was no left ventricular or RV hypertrophy. CMR = cardiac magnetic resonance imaging; RV = right ventricle.

An initial CT pulmonary angiogram (CTPA) showed no pulmonary embolism but revealed multiple bilateral lung lesions, including one that appeared to extend anteriorly toward the right ventricle (RV). A subsequent transesophageal echocardiogram showed a mobile cystic mass measuring 2.4 × 2.0 cm and a separate solid mass measuring 1.0 × 0.9 cm, both attached to the RV free wall (Figures 2A and 2B, Video 1) before the CTPA (Figure 3). Cardiac magnetic resonance imaging (CMR) confirmed a multicystic mass attached to the RV free wall, without evidence of RV dysfunction (Figures 4A to 4C).

Upon referral to a tertiary hospital for evaluation of the cardiac mass, the patient continued to report cough, shortness of breath on exertion, and hemoptysis. Respiratory examination showed normal breath sounds without stridor, respiratory distress, or wheezing. He had a left minithoracotomy scar. Cardiac examination revealed normal heart sounds and a regular sinus rhythm. Chest radiography showed multiple bilateral lung nodules.

Laboratory test results were negative for sputum acid-fast bacilli and serum antineutrophil cytoplasmic antibodies. Complete blood count showed a white blood count of 12.4 × 10^9^/L, neutrophils 7.8 × 10^9^/L, eosinophils 1.38 × 10^9^/L, C-reactive protein 6.4 mg/L, and an activated partial thromboplastin time of 48 seconds. Serologic test findings were positive for antibodies against *Echinococcus*, *Strongyloides*, and *Cysticercosis* and negative for *Schistosoma* (bilharziasis), *Aspergillus*, and HIV.

## Management (Medical/Interventions)

Owing to the rare presentation of a hydatid cyst in the heart, the diagnosis was initially missed on the CTPA. However, in combination with echocardiography, a subsequent review of the CT scan revealed an RV cardiac mass, prompting immediate cardiology consultation. The patient was started on a therapeutic dose of enoxaparin for 1 day because of a concern for a cardiac thrombus, despite ongoing hemoptysis and melena. Anticoagulation was discontinued after CMR findings were not consistent with thrombus.

After consultation with the infectious diseases team, therapy for disseminated *Echinococcus* infection was initiated on the basis of lung biopsy findings and positive serology test results. The patient was started on albendazole 400 mg twice daily and praziquantel 1200 mg 3 times daily. Once investigations confirmed bilateral pulmonary lesions and RV cystic lesions, surgical resection was planned. Preoperative management included at least 14 days of albendazole therapy along with prophylactic enoxaparin for deep vein thrombosis prevention.

### Surgical operative notes

1. Bilateral upper-lobe wedge resections were performed by the thoracic surgery team.
2. Resection of the intracardiac mass (RV cyst excision) was performed by the cardiac surgery team.

### Thoracic surgery

A full-length median sternotomy was performed, followed by exploration of both pleural cavities. Wedge resections of the most medial cysts in the left and right upper lobes were completed.

### Cardiac surgery

**Figure 5:**
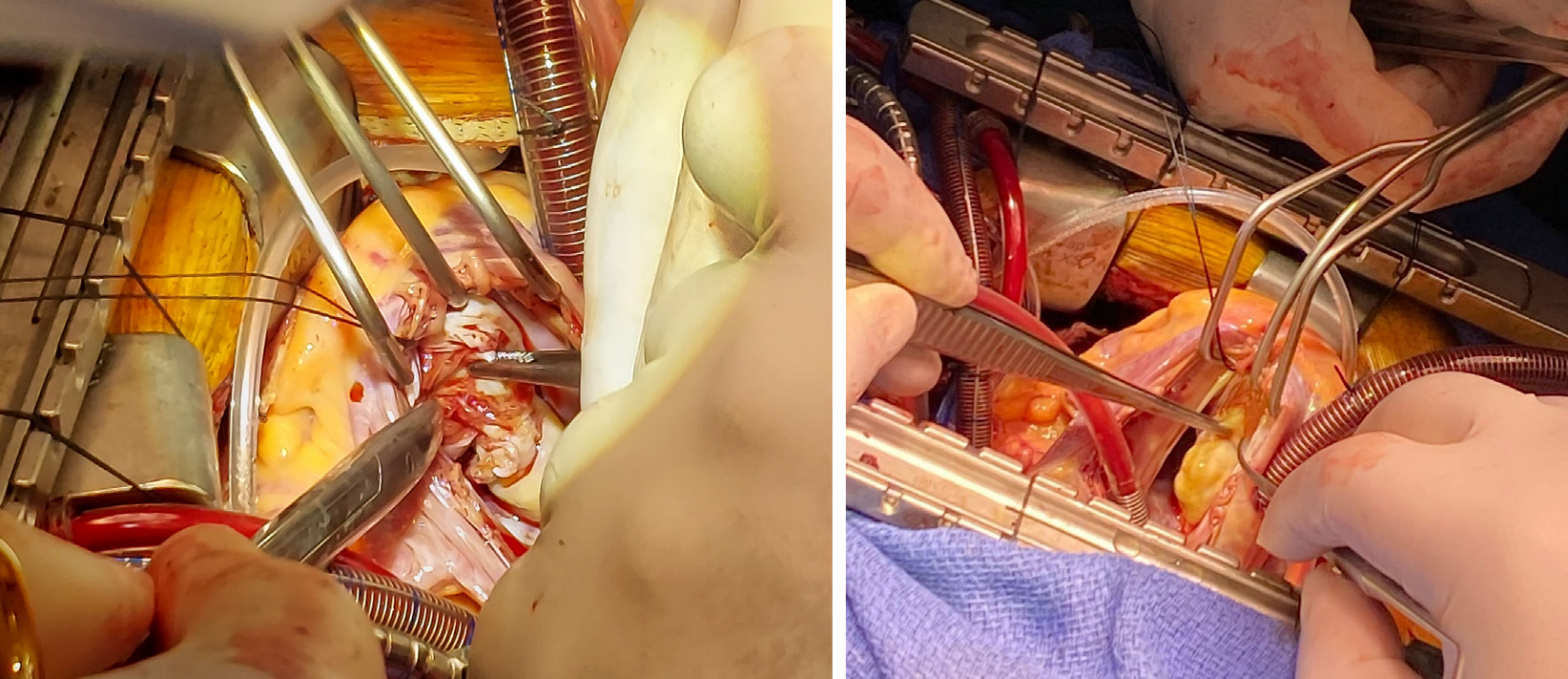
Intraoperative Images of Cardiac Cyst Resection (A and B) Intraoperative photographs of the surgical resection of the right ventricular cardiac cyst mass. Reproduced with kind permission of Dr Gopal Bhatnagar, Chief of Cardiac Surgery, Cleveland Clinic Abu Dhabi.

Midline sternotomy was performed, and cardiopulmonary bypass was established with cardioplegia infusion. Adequate asystole was achieved. Through a right atrial incision, the tricuspid valve was retracted, and the cyst was identified and carefully dissected from the myocardium using sharp and blunt techniques. The area was irrigated with hypertonic saline. After resection, the tricuspid valve was tested and found to be intact (Figures 5A and 5B). The atrium was closed in 2 layers.

## Outcome and Follow-Up

### Postoperative transesophageal echocardiogram

The left ventricle was normal in size and wall thickness, with preserved systolic function. The left ventricular ejection fraction was 60%. The RV was normal in size but showed mild dysfunction. Trivial mitral and tricuspid regurgitations were noted.

### Postsurgery progress

The patient remained hemodynamically stable with no complications. Electrocardiography showed no significant changes. Follow-up transthoracic echocardiography (TTE) showed mild RV dilation with moderately reduced RV systolic function. Fractional area change was 32%. Mild tricuspid regurgitation was present. Estimated RV systolic pressure was 67 mm Hg, consistent with pulmonary hypertension. Interval development of RV dilation and severe pulmonary hypertension were noted, attributed to postoperative fluid overload. The pulmonary hypertension subsequently improved.

## Discussion

Human cystic echinococcosis (CE), also known as hydatid disease, affects more than 1 million individuals annually, according to the World Health Organization. It is endemic in Mediterranean countries, the Middle East, South America, and Australia.^1^ CE is caused by infection with the larvae of *Echinococcus granulosus*, a parasite whose definitive hosts are carnivores such as dogs, with sheep serving as intermediate hosts and humans as accidental hosts.

After ingestion, the oncospheres of the parasite penetrate the duodenal mucosa and enter the venous or lymphatic system, allowing access to multiple organs.^1^ *E. granulosus* most commonly affects the liver (65%) and lungs (25%). Cardiac involvement is rare, with a reported prevalence of 0.5% to 2%.^2^ Furthermore, isolated cardiac involvement is uncommon because infection typically occurs secondary to involvement of other organs.

The rarity of cardiac involvement is attributed to filtering functions of the liver and lungs, as well as the continual contraction of the cardiac wall, which limits cyst implantation.^2^ When the parasite does adhere to the myocardium, the disease may localize to the left ventricle (60%), RV (10%), pericardium (7%), left atrium (6%-8%), right atrium (3%-4%), or the interventricular septum (4%).^3^ The left ventricle is most commonly affected because of its thick musculature and rich vascular supply.^4^ After implantation, the eggs gradually develop into mature hydatid cysts, growing at a rate of approximately 1 cm per year. In our patient, a cystic mass (2.4 × 2.0 cm) and a solid mass (1.0 × 0.9 cm) were identified on the RV free wall.

RV involvement can be especially critical because of the risk of intracavity rupture and obstruction of the valvular orifice, which may result in anaphylactic shock, pulmonary embolism, or death in up to 30% of cases.^5^

The clinical presentation of cardiac hydatid disease varies. Reported symptoms in the literature include chest pain, palpitations, atrioventricular block, dyspnea or syncope, cardiac tamponade, pulmonary hypertension, and cardiac murmur. Unless acute complications occur, patients typically present with chronic, nonspecific symptoms because of the slow progression of cyst development. In our case, the patient's primary symptoms were chronic hemoptysis, melena, and weight loss. His melena may have resulted from swallowing increased volumes of bloodied cough.

Electrocardiographic changes are typically nonspecific, with normal sinus rhythm being the most commonly observed finding.^6^ Imaging modalities that have proven useful include TTE, often supplemented by CT or CMR. On TTE, cystic CE lesions typically appear echolucent but may become solid or hyperechogenic over time because of calcification, solidification, or degeneration.^7^ As a result, the differential diagnosis for cardiac hydatid cysts includes cardiac blood cyst, thrombus, and tumors—conditions that were initially favored over hydatid disease in our case.

CT imaging can aid in distinguishing cysts from solid tumors such as myxomas or fibromas, and CMR is valuable for ruling out other cystic cardiac masses. Cardiac hydatid cysts have distinctive imaging features, such as calcification of the cyst wall, membrane detachment, and the presence of daughter cysts. In our patient, investigations included TTE, CT, and CMR. Unfortunately, the initial CTPA did not clearly demonstrate cardiac involvement, which delayed the use of echocardiography (Video 2). CMR ultimately served as the key modality for imaging confirmation after TTE.

The mainstay of laboratory diagnosis for CE is serologic testing, with indirect hemagglutination and enzyme-linked immunosorbent assay (ELISA) being the most commonly used methods.

The burden of echinococcosis can be reduced with albendazole treatment before surgery; however, surgical resection remains the definitive treatment for cardiac CE. Unlike abdominal CE cysts, cardiac cysts are typically resected regardless of whether they are in an active or inactive stage. Intraoperatively, the pulmonary artery is often clamped to prevent embolization, and meticulous cystectomy is performed to avoid rupture and the risk of anaphylactic shock. Hypertonic saline was used as the protoscolicidal agent during surgery.^6^ The duration of postoperative albendazole therapy typically ranges from 2 weeks to 4 months.^8^^,^^9^

## Conclusions

Cardiac involvement by the *Echinococcus* parasite is rare and presents a significant diagnostic challenge because of its complex and often nonspecific clinical presentation. In our case, the patient's symptoms of hemoptysis and weight loss did not immediately raise suspicion of cardiac disease, especially given the uncommon geographical occurrence of cardiac echinococcosis. The initial CTPA failed to detect cardiac involvement, and echocardiography was not performed until 1 month after admission—an identified weakness in the diagnostic approach.

Delays in diagnosis and surgical intervention can increase health care burden through repeated hospital visits, persistent symptoms, and patient distress. In cases where metastatic disease and other chronic inflammatory conditions have been excluded, parasitic infection should remain a consideration. In our patient, CMR proved essential for diagnosis, and biopsy with histopathology confirmed the final diagnosis.

## Funding Support and Author Disclosures

The authors have reported that they have no relationships relevant to the contents of this paper to disclose.

## Appendix

Video 1 Trans esophageal echocardiogram showing right ventricular hydatid cyst attached to the free wall, mobile during cardiac cycle.

Video 2 XXX
